# Role of Nanotechnology in Cosmeceuticals: A Review of Recent Advances

**DOI:** 10.1155/2018/3420204

**Published:** 2018-03-27

**Authors:** Shreya Kaul, Neha Gulati, Deepali Verma, Siddhartha Mukherjee, Upendra Nagaich

**Affiliations:** Department of Pharmaceutics, Amity Institute of Pharmacy, Amity University, Noida, Uttar Pradesh 201301, India

## Abstract

Nanotechnology manifests the progression in the arena of research and development, by increasing the efficacy of the product through delivery of innovative solutions. To overcome certain drawbacks associated with the traditional products, application of nanotechnology is escalating in the area of cosmeceuticals. Cosmeceuticals are regarded as the fastest growing segment of the personal care industry and the use has risen drastically over the years. Nanocosmeceuticals used for skin, hair, nail, and lip care, for conditions like wrinkles, photoaging, hyperpigmentation, dandruff, and hair damage, have come into widespread use. Novel nanocarriers like liposomes, niosomes, nanoemulsions, microemulsion, solid lipid nanoparticles, nanostructured lipid carrier, and nanospheres have replaced the usage of conventional delivery system. These novel nanocarriers have advantages of enhanced skin penetration, controlled and sustained drug release, higher stability, site specific targeting, and high entrapment efficiency. However, nanotoxicological researches have indicated concern regarding the impact of increased use of nanoparticles in cosmeceuticals as there are possibilities of nanoparticles to penetrate through skin and cause health hazards. This review on nanotechnology used in cosmeceuticals highlights the various novel carriers used for the delivery of cosmeceuticals, their positive and negative aspects, marketed formulations, toxicity, and regulations of nanocosmeceuticals.

## 1. Introduction

Nanotechnology is regarded as the most imminent technology of 21st century and is contemplated as a big boon in the cosmetic industry. The term nanotechnology is the combination of two words: namely, technology and the Greek numerical “nano” which means dwarf. Thus, nanotechnology is considered as the science and technology used to develop or manipulate the particles in the size range of 1 to 100 nm [1, 2]. Since 1959, nanotechnology has emerged in different fields like engineering, physics, chemistry, biology, and science and it has been virtually 40 years since nanotechnology has intruded into the field of cosmetics, health products, and dermal preparations. During the era of 4000BC, the use of nanotechnology has been recorded by the Egyptians, Greek, and Romans, with concept of hair dye preparation utilizing nanotechnology [3].

Founding member of US society of Cosmetic Chemists, Raymond Reed, coined the term “cosmetics” in the year 1961. Cosmetics can be defined as the products which amplify the appearance of the skin, intensify the cleansing, and promote the beauty of the skin [4]. As reported, the use of cosmetics was attributed to Egyptians around 4000BC and later Greeks, Romans, Chinese, Japanese, and Americans started using cosmetics. In the late 19th century, the use of cosmetics was secretly done by the women with household items in western countries and by 20th century the cosmetics were being done without concealment. By the 21th century, the cosmetics are being enormously used and with the development in technology, innovative cosmetic formulations are being developed by the incorporation of the latest technologies [5, 6].

Cosmeceuticals are the cosmetic products which incorporate biologically active ingredient having therapeutic benefits on the surface applied. These are utilized as cosmetics as they claim to enhance appearance [7]. Cosmeceuticals are chasm between pharmaceuticals and personal care products. Cosmeceutical products have measurable therapeutic efficacy on the skin, as drugs and formulations have diversified from skin to body to hair and they are used for the treatment of various conditions like hair damage, wrinkles, photoaging, skin dryness, dark spots, uneven complexion, hyperpigmentation, and so on [8].

Cosmeceuticals are contemplated as the fastest growing fragment of personal care industry and the market for personal care is increasing enormously [9]. Despite enormous benefits of nanoparticles, little is known about the short-term and long-term health effects in the environment and organisms. Safety concerns have been raised due to the reported toxicity and possible dangers of the nanomaterials. The present article reviews the diverse classes of nanocarriers like liposomes, niosomes, solid lipid nanoparticles, nanostructured lipid carriers, nanoemulsion, and so on which are being used for delivery of nanocosmeceuticals, marketed products, and positive and negative aspects.

**Figure 1:**
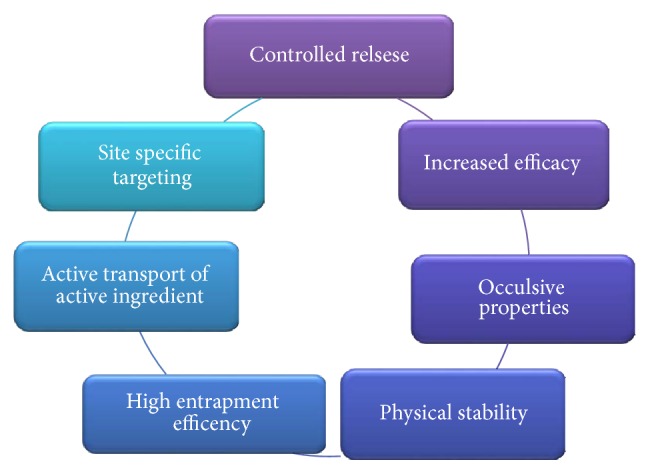
Pictorial presentation of positive aspects of nanocosmeceuticals.

There are a number of advantages of nanocosmeceuticals. Namely, they provide the controlled release of active substances by controlling the drug release from carriers by several factors including physical or chemical interaction among the components, composition of drug, polymer and additives, ratio, and preparation method. They are used in hair care preparations, such as in treatment of hair loss and to prevent hair from turning grey such as Identik Masque Floral Repair, Origem hair recycling shampoo, and Nirvel hair-loss control shampoo. Nanocosmeceuticals make the fragrances last longer, for example, Allure Parfum and Allure Eau Parfum spray by Chanel. These make the skin care formulations more effective and increase the efficacy of sunscreens by improving UV protection in them. By having very small size of the particles, the surface area is increased which allows the active transport of the active ingredients into the skin. Occlusion provides the enhancement in the penetration and skin hydration is increased. Cosmeceuticals have high entrapment efficiency and good sensorial properties and are more stable than the conventional cosmetics. Most of the nanoparticles are suitable for both lipophilic and hydrophilic drug delivery. Nanomaterials are widely used in the preparation of antiwrinkle creams, moisturizing creams, skin whitening creams, hair repairing shampoos, conditioners, and hair serums [11, 12]. Several positive aspects of nanocosmeceuticals are discussed in Figure 1 [13].

**Figure 2:**
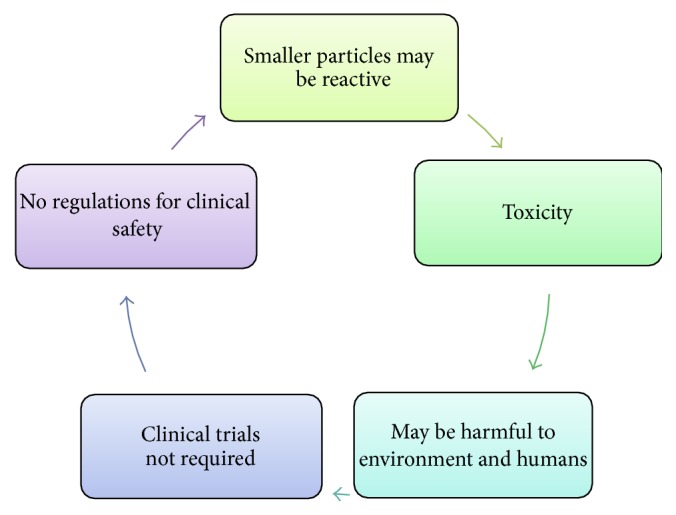
Pictorial presentation of negative aspects of nanocosmeceuticals.

As the rule of the nature, each and everything in this universe has some positive as well as negative aspects. Some of the drawbacks associated with nanocosmeceuticals are as follows. Due to production of large number of oxygen species, oxidation stress, inflammation, damage to DNA, proteins, and membranes may be caused by nanoparticles. Few ultrafine nanomaterials such as carbon nanotubes, carbon based fullerenes, TiO_2_, copper nanoparticles, and silver nanoparticles may be toxic to human tissues and cells. Titanium dioxide found in sunscreens has been demonstrated to cause damage to DNA, RNA, and fats within cells. No stringent scrutiny was imposed by the regulatory agencies for the approval and regulation of nanocosmeceuticals. Nanocosmeceuticals may be harmful to environment as well. No clinical trials are required for the approval of nanocosmeceuticals, thus raising a concern of toxicity after use [15, 16]. Negative aspects of nanocosmeceuticals are discussed in Figure 2 [17].

## 2. Novel Nanocarriers for Cosmeceuticals

**Figure 3:**
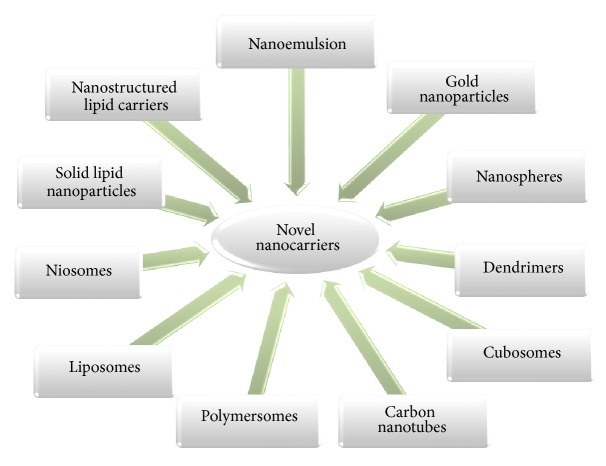
Pictorial presentation of various nanocarriers for cosmeceuticals.

For the delivery of nanocosmeceuticals, carrier technology is employed which offers an intelligent approach for the delivery of active ingredients. Various novel nanocarriers for delivery of cosmeceuticals are depicted in Figure 3 [18, 19].

### 2.1. Liposomes

Liposomes are most widely used for the cosmeceutical preparations. They are the vesicular structures having an aqueous core which are enclosed by a hydrophobic lipid bilayer [20]. The main component of liposome lipid bilayer is phospholipids; these are GRAS (generally recognized as safe) ingredients, therefore minimizing the risk for adverse effects [21]. To protect the drug from metabolic degradation, liposome encapsulates the drug and releases active ingredients in a controlled manner [22]. Liposomes are suitable for delivery of both hydrophobic as well as hydrophilic compounds. Their size varies from 20 nm to several micrometers and can have either multilamellar or unilamellar structure [23].

Antioxidants such as carotenoids, CoQ10, and lycopene and active components like vitamins A, E, and K have been incorporated into liposomes, which are used to amplify their physical and chemical stability when dispersed in water [24].

Phosphatidylcholine is the key component of liposomes which has been used in various skin care formulations like moisturizer creams and so on and hair care products like shampoo and conditioner due to its softening and conditioning properties. Due to their biodegradable, nontoxic, and biocompatible nature, liposomes are used in variety of cosmeceuticals as they encapsulate active moiety [25]. Vegetable phospholipids are widely used for topical applications in cosmetics and dermatology because of their high content of esterified essential fatty acids. For topical applications in cosmetics and dermatology vegetable phospholipids are being widely used as they have high content of esterified essential fatty acids. After the application of linoleic acid, within a short period of time the barrier function of the skin is increased and water loss is decreased. Vegetable phospholipids and soya phospholipids are used because of their ability to form liposomes and their surface activity. The transport of linoleic acid into the skin is done by these phospholipids [26, 27]. In a clinical study, it was proven that flexible liposomes help in wrinkle reduction and show effects like decreasing of efflorescence in the acne treatment and an increase in skin smoothness [28].

**Figure 4:**
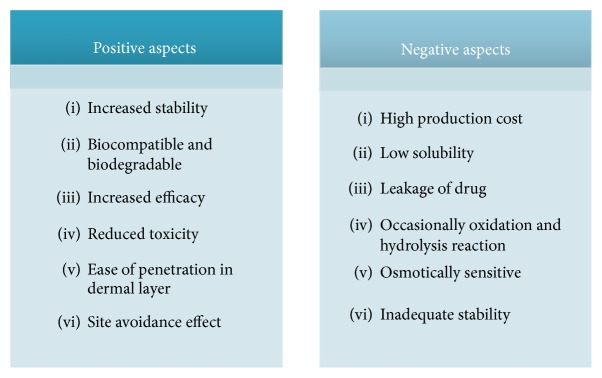
Pictorial presentation of positive aspects of liposomes.

Liposomes are being developed for the delivery of fragrances, botanicals, and vitamins from anhydrous formulations, such as antiperspirants, body sprays, deodorants, and lipsticks. They are also being used in antiaging creams, deep moisturizing cream, sunscreen, beauty creams, and treatment of hair loss [29]. Several positive and negative aspects of liposomes are discussed in Figure 4 [30]. Various marketed formulations are given in Table 1 [31–33].

### 2.2. Niosomes

Niosomes are defined as vesicles having a bilayer structure that are made up by self-assembly of hydrated nonionic surfactants, with or without incorporation of cholesterol or their lipids [34].

Niosomes can be multilamellar or unilamellar vesicles in which an aqueous solution of solute and lipophilic components are entirely enclosed by a membrane which are formed when the surfactant macromolecules are organized as bilayer [35]. Size ranges from 100 nm to 2 *μ*m in diameter. Size of small unilamellar vesicles, multilamellar vesicles, and large unilamellar vesicles ranges from 0.025–0.05 *μ*m, =>0.05 *μ*m, and 0.10 *μ*m, respectively [36]. Major niosomes components include cholesterol and nonionic surfactants like spans, tweens, brijs, alkyl amides, sorbitan ester, crown ester, polyoxyethylene alkyl ether, and steroid-linked surfactants which are used for its preparation [37].

Niosomes are suitable for delivery of both hydrophobic as well as hydrophilic compounds. As a novel drug delivery system, niosomes can be used as vehicle for poorly absorbable drugs [38]. It provides encapsulation to the drug, due to which the drug in the systemic circulation is for prolonged period and penetration is enhanced into target tissue. Niosomes overcome the problems associated with liposomes, like stability problems, high price, and susceptibility to oxidation [39]. Niosomes are used in cosmetics and skin care applications since skin penetration of ingredients is enhanced because it possesses the property of reversibly reducing the barrier resistance of the horny layer, allowing the ingredient to reach the living tissues at greater rate. There is increased stability of entrapped ingredients and improvement in the bioavailability of poorly adsorbed ingredients. There are many factors affecting formation of niosomes, namely, nature and structure of surfactants, nature of encapsulated drug, membrane composition, and temperature of hydration which influences shape and size [40]. Specialized niosomes are called proniosomes; these are nonionic based surfactant vesicles, which are hydrated immediately before use to yield aqueous niosome dispersions. To enhance drug delivery in addition to conventional niosomes, proniosomes are also being used [41, 42].

**Figure 5:**
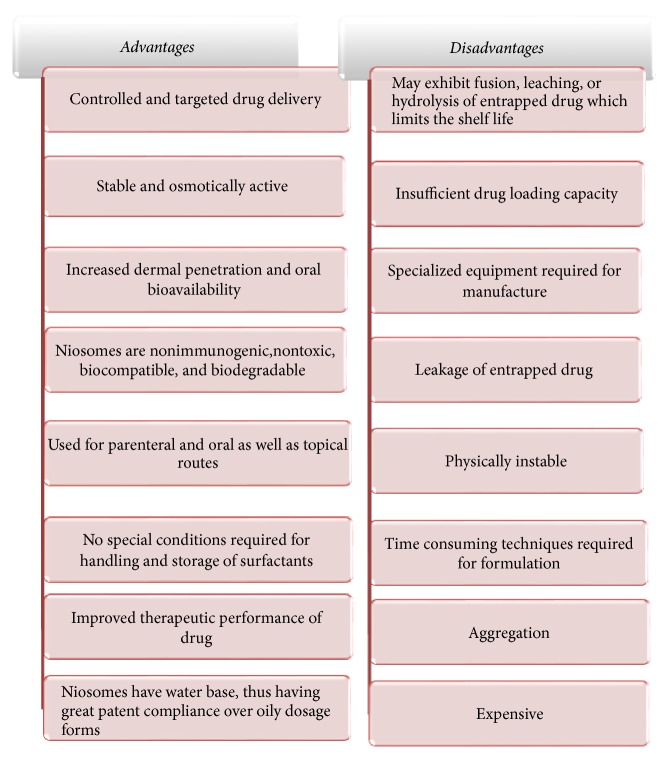
Advantages and disadvantages of niosomes.

Niosomes were first developed by L'Oreal in the year 1970 by the research and development of synthetic liposomes. Niosomes were patented by L'Oreal in the year 1987 and were developed under the trade name of Lancome. Various niosomes cosmeceuticals preparations are available in market, antiwrinkle creams, skin whitening and moisturizing cream, hair repairing shampoo, and conditioner [43]. Several advantages and disadvantages of niosomes are discussed in Figure 5 [44–46]. Various marketed products and uses are discussed in Table 2 [47–49].

### 2.3. Solid Lipid Nanoparticles

An unconventional carrier system, solid lipid nanoparticle (SLN), was developed at the beginning of the 1990s, over the conventional lipoidal carriers like emulsions and liposomes. 50 to 1000 nm is the size range of solid lipid nanoparticles [50].

They are composed of single layer of shells and the core is oily or lipoidal in nature. Solid lipids or mixtures of lipids are present in the matrix drug which is dispersed or dissolved in the solid core matrix. Phospholipids hydrophobic chains are embedded in the fat matrix. These are prepared from complex glycerides mixtures, purified triglycerides, and waxes; liquid lipid is replaced by solid lipid or blend of solid lipid which is solid at body and room temperature and is stabilized by surfactants or polymers [51]. Lipophilic, hydrophilic, and poorly water-soluble active ingredients can be incorporated into SLNs which consist of physiological and biocompatible lipids. With the use of biocompatible compounds for preparing SLN, toxicity problems are avoided [52]. Two principle methods for preparation of SLNs are high pressure homogenization method and precipitation method. Controlled release and sustained release of the active ingredients are possible; SLN which has drug enriched core leads to a sustained release and SLN having drug enriched shell shows burst release [53, 54].

SLNs are popular in cosmeceuticals and pharmaceuticals as they are composed of biodegradable and physiological lipids that exhibit low toxicity. Their small size ensures that they are in close contact with the stratum corneum which increases the penetration of active ingredients through the skin [55].

SLNs have UV resistant properties and act as physical sunscreens on their own, so improved photoprotection with reduced side effects can be achieved when they are combined with molecular sunscreen [56]. Solid lipid nanoparticles as carrier for 3,4,5-trimethoxybenzoylchitin and vitamin E sunscreen are developed to enhance UV protection [57]. SLNs have occlusive property which can be used to increase the skin hydration, that is, water content of the skin [58]. Perfume formulations also have SLNs as they delayed the release of perfume over longer period of time and are ideal for use in day creams as well [59, 60].

**Figure 6:**
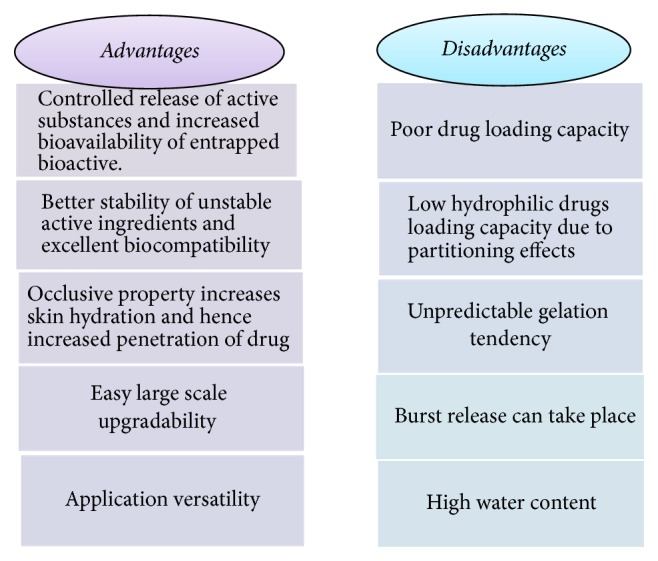
Benefits and drawbacks of SLNs.

They have better stability coalescence when compared to liposomes because they are solid in nature and mobility is reduced of the active molecules, so the leakage from the carrier is prevented [61, 62]. Benefits and drawbacks of SLNs are depicted in Figure 6 [50, 63–65]. Different marketed products and their uses are given in Table 3 [66, 67].

### 2.4. Nanostructured Lipid Carriers (NLC)

Nanostructured lipid carriers are considered as the second generation of the lipid nanoparticles. NLC have been developed so that the drawbacks associated with SLN can be overcome. NLC are prepared through blending by solid lipids along with spatially incompatible liquid lipid leading to amorphous solids in preferable ratio of 70 : 30 up to 99.9 : 0.1 being solid at body temperature [68, 69]. NLC are mainly of three types on the basis of which the structure is developed according to the formulation composition and production parameters, namely, imperfect type, amorphous type and multiple type. The particle size ranges from 10 to 1000 nm [70].

There is an increased scientific and commercial attention for NLC during the past few years because of the lower risk of systemic side effects. NLC when compared to SLN show higher drug-loading capacity for entrapped bioactive compound because of the distorted structure which helps in creating more space. Other limitations of SLN like reducing particle concentration and expulsion of drug during storage are solved by the formulation of NLC. They are formulated by biodegradable and physiological lipids that show very low toxicity [71]. NLC have modulated drug delivery profile, that is, a biphasic drug release pattern; in this the drug is released initially with a burst followed by a sustained release at a constant rate. They possess numerous advantageous features like increased skin hydration due to occlusive properties, and the small size ensures close contact to the stratum corneum leading to the increased amount of drug penetration into the skin. There are stable drug incorporation during storage and enhanced UV protection system with reduced side effects [72].

**Figure 7:**
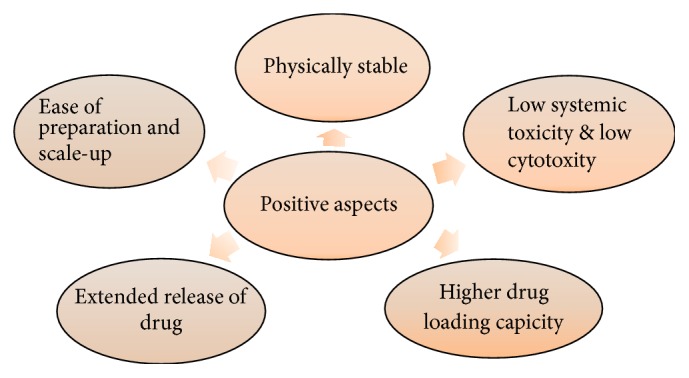
Various positive aspects depiction of NLC.

In October 2005, the first products containing lipid nanoparticles were introduced in the cosmetic market, namely, NanoRepair Q10® cream and NanoRepair Q10® serum, Dr. Rimpler GmbH, Germany, offering increased skin penetration. Currently there are more than 30 cosmetic products available in the market containing NLC [73, 74]. Depiction of various positive aspects of NLC is depicted in Figure 7 [75, 76]. List of marketed products, manufacturers, and their uses is given in Table 4 [77–80].

### 2.5. Nanoemulsions

Nanoemulsions are considered as the kinetically or thermodynamically stable dispersion of liquid in which an oil phase and water phase are in combination with a surfactant. Their structure can be manipulated on the basis of method of preparation, so as to give different types of products. Depending on the composition different types of nanoemulsions are oil in water, water in oil, and bicontinuous nanoemulsion. They exhibit various sizes ranging from 50 nm to 200 nm. These are dispersed phase which comprises small particles or droplets, having very low oil/water interfacial tension [81]. They have lipophilic core, which is surrounded by a monomolecular layer of phospholipids, making it more suitable for delivery of lipophilic compounds.

Problems like sedimentation, coalescence, creaming, and flocculation are not associated with nanoemulsions like with macromolecules. Nanoemulsions are transparent or translucent and show properties like low viscosity, high kinetic stability, high interfacial area, and high solubilization capacity [82].

Nanoemulsions are widely used as medium for the controlled delivery of various cosmeceuticals like deodorants, sunscreens, shampoos, lotions, nail enamels, conditioners, and hair serums [83].

**Figure 8:**
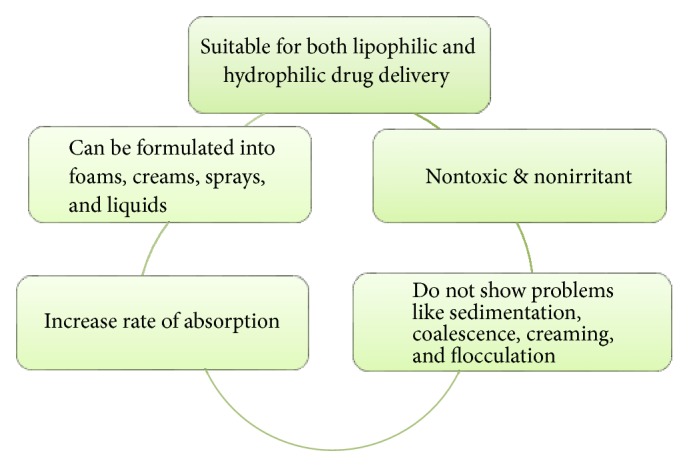
Merits of nanoemulsions.

In cosmetics formulation, nanoemulsions provide rapid penetration and active transport of active ingredients and hydration to the skin. Merits of nanoemulsion are shown in Figure 8 [84–87]. Various marketed products name, manufacturers, and their uses are given in Table 5 [88–90].

### 2.6. Gold Nanoparticles

Nanogold or gold nanoparticles exhibit various sizes ranging from 5 nm to 400 nm. Interparticle interactions and assembly of gold nanoparticles play an important role in determination of their properties [91]. They exhibit different shapes such as nanosphere, nanoshell, nanocluster, nanorod, nanostar, nanocube, branched, and nanotriangles. Shape, size, dielectric properties, and environmental conditions of gold nanoparticles strongly affect the resonance frequency. The color of nanogold ranges from red to purple, to blue and almost black due to aggregation [92]. Gold nanoparticles are inert in nature, highly stable, biocompatible, and noncytotoxic in nature. Nanogold is very stable in liquid or dried form and is nonbleaching after staining on membranes; they are also available in conjugated and unconjugated form [93]. They have high drug-loading capacity and can easily travel into the target cell due to their small size and large surface area, shape, and crystallinity [94].

**Figure 9:**
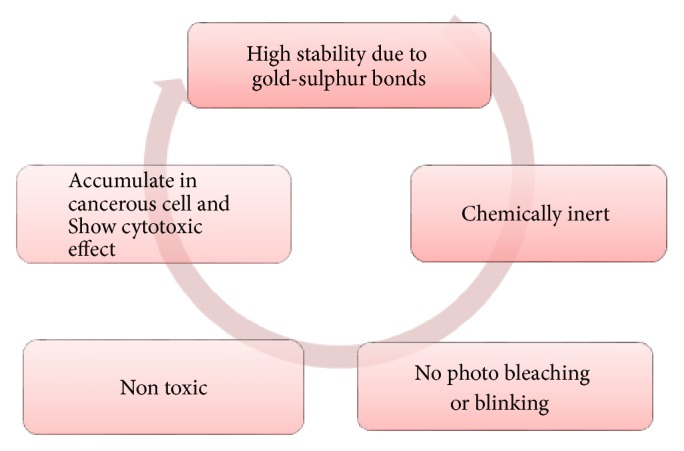
Description of merits of gold nanoparticles.

Gold nanoparticles have been studied as a valuable material in cosmeceutical industries due to their strong antifungal and antibacterial properties. These nanoparticles are used in variety of cosmeceuticals products like cream, lotion, face pack, deodorant, antiaging creams, and so forth. Cosmetic giant companies like L'Oreal and L'Core Paris are using gold nanoparticles for manufacturing more effective creams and lotions [95]. Main properties of nanogold in beauty care consist of assets, namely, acceleration of blood circulation, anti-inflammatory property, antiseptic properties, improvising firmness and elasticity of skin, delaying aging process, and vitalizing skin metabolism [96]. Description of merits of gold nanoparticles is depicted in Figure 9 [97–99]. List of various marketed products name, manufacturers, and their uses is given in Table 6 [100–104].

### 2.7. Nanospheres

Nanospheres are the spherical particles which exhibit a core-shell structure. The size ranges from 10 to 200 nm in diameter. In nanospheres, the drug is entrapped, dissolved, attached, or encapsulated to the matrix of polymer and drug is protected from the chemical and enzymatic degradation. The drug is physically and uniformly dispersed in the matrix system of polymer. The nature of the nanospheres can be crystalline or amorphous [105]. This system has great potential and is being able to convert poorly absorbed, labile biologically active substance and poorly soluble active substance into the propitious deliverable drug. The core of nanospheres can be enclosed with diverse enzymes, genes, and drugs [106].

Nanospheres can be divided into two categories: biodegradable nanospheres and nonbiodegradable nanospheres. Biodegradable nanospheres include gelatin nanospheres, modified starch nanospheres, and albumin nanospheres and nonbiodegradable nanospheres include polylactic acid, which is the only approved polymer.

**Figure 10:**
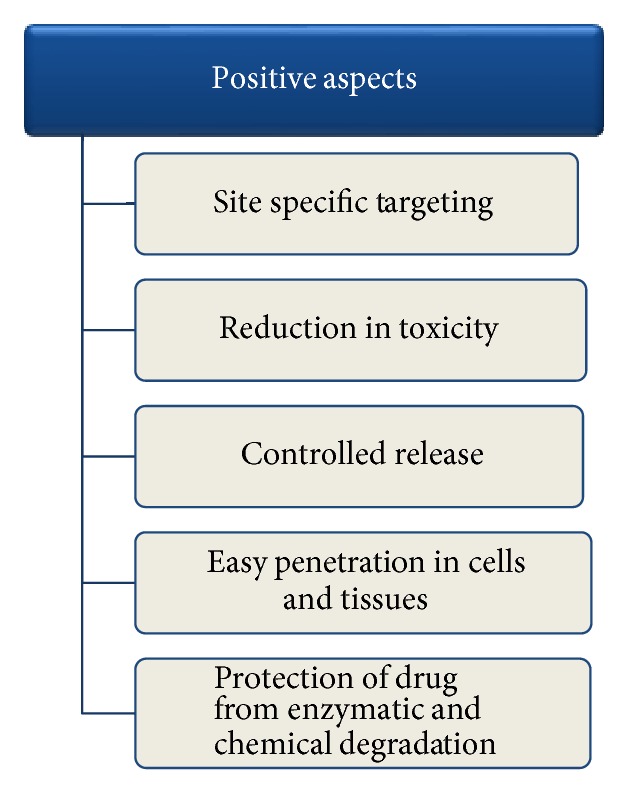
Favorable aspects of nanospheres [14].

In cosmetics, nanospheres are used in skin care products to deliver active ingredients into deep layer of the skin and deliver their beneficial effects to the affected area of the skin more precisely and efficiently. These microscopic fragments play a favorable role in protection against actinic aging. Use of nanospheres is increasing in the field of cosmetics especially in skin care products like antiwrinkle creams, moisturizing creams, and antiacne creams [107]. Pictorial presentation of favorable aspects of nanospheres is depicted in Figure 10 [14]. Marketed products name, manufacturers, and their uses are given in Table 7 [10].

### 2.8. Dendrimers

The term “dendrimer” arises from two Greek words: namely, “Dendron” that means tree and “Meros” meaning part. Dendrimers are highly branched, unimolecular, globular, micellar nanostructure, and multivalent nanoparticles whose synthesis theoretically affords monodisperse compounds. A dendrimer is typically built from a core on which one or a number of successive series of branches are engrafted in an arborescent way and often adopts a spherical three-dimensional morphology [108]. Generation of the dendrimer is determined by total number of series of branches: if it has one series of branches, then it is first-generation dendrimer; if it has two series, then it is second generation and so on. They are extremely small in size, having diameters in the range of 2–20 nm [109]. Its other properties like monodispersity, polyvalence, and stability make it an ideal carrier for drug delivery with precision and selectivity. To attach biologically active substances for targeting purpose terminal groups are modified. Dendrimers provide controlled release from the inner core and drugs are incorporated in interior as well as being attached on the surface [110].

**Figure 11:**
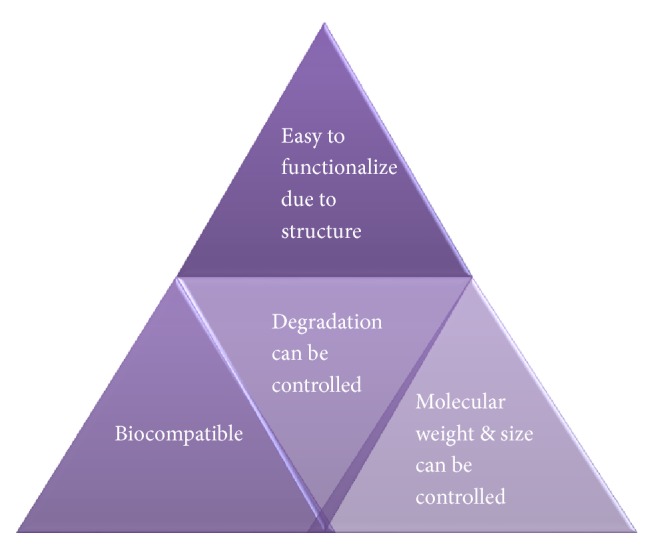
Advantages of dendrimers.

Dendrimers are new class of macromolecular architecture and are also being used as nanotechnology based cosmeceuticals for various applications like in hair care, skin care, and nail care. Dendrimers have utility in various cosmetic products like shampoos, sunscreen, hair-styling gels, and antiacne products [111]. Companies like L'Oreal, The Dow Company, Wella, and Unilever have several patents for application of dendrimers in cosmeceuticals. Descriptions of advantages of dendrimers are represented in Figure 11.

### 2.9. Carbon Nanotubes

In the field of nanotechnology, carbon nanotubes are represented as one of the most unique inventions. Carbon nanotubes (CNTs) can be described as the rolled graphene with SP^2^ hybridization. These are seamless cylindrical hollow fibers, comprised of walls formed by graphene as hexagonal lattice of carbon, which are rolled at specific and discrete “chiral” angles. Individual carbon nanotubes align themselves naturally into “ropes” held together by pi-stacking. The diameter ranges from 0.7 to 50 nm with lengths in the range of 10's of microns [112, 113]. Carbon nanotubes are extremely light in weight. These are further of 3 types: namely, single-walled CNTs, doubled-walled CNTs, and multiwalled CNTs. Single-walled CNTs are made up of single graphene sheet which is rolled upon itself with diameter of 1-2 nm, double-walled CNTs are made of two concentric carbon nanotubes, and multiwalled CNTs consist of multiple layers of graphene tubes having diameter ranging from 2 to 50 nm [114]. Major production methods of carbon nanotubes consist of arc discharge method, laser ablation, chemical vapor deposition method, flame synthesis method, and silane solution method [115]. Various patents of carbon nanoparticles have been filed in the field of cosmeceuticals like hair coloring and cosmetic compositions comprising carbon nanotubes and peptide-based carbon nanotube hair colorants and their use in hair colorant and cosmetic compositions [116, 117].

### 2.10. Polymersomes

Polymersomes are artificial vesicles which enclose a central aqueous cavity, composed of self-assembly of block copolymer amphiphiles. They have a hydrophilic inner core and lipophilic bilayer, so they can be used for both lipophilic and hydrophilic drugs and hydrophobic core provides a protein-affable environment [118]. Polymersomes are biologically stable and are highly versatile. Their drug encapsulation and release capabilities can be easily modulated by applying various block copolymers that are biodegradable or stimuli-responsive. There radius ranges from 50 nm to 5 *μ*m or more [119]. Polymersomes are proficient for encapsulating and protecting sensitive molecules, namely, drugs, proteins, peptides, enzymes, DNA, and RNA fragments. For the preparation of polymersomes, generally synthetic block copolymers have been used. The composition and molecular weight of these polymers can be varied, which allows preparation of polymersomes with different properties, responsiveness to stimuli, different membrane thickness, and permeabilities [120, 121]. The flexibility in the membrane of polymersome makes them capable of targeting and controlling drug release. Due to the presence of a thick and rigid bilayer, they offer more stability than liposomes [122]. Polymersomes are being investigated in the cosmeceutical industry for their use and various patents have been filed for their use. Patents have been filed using polymersome for improving skin elasticity and in another patent polymersomes are used for skin cell activation energy enhancement [123, 124].

### 2.11. Cubosomes

Cubosomes are the advanced nanostructured particles which are discrete, submicron, and self-assembled liquid crystalline particles of surfactants with proper ratio of water that provides unique properties. Cubosomes are formed by self-assembled structures of aqueous lipid and surfactant systems when mixed with water and microstructure at a certain ratio [125]. Cubosomes are bicontinuous cubic liquid phase, which encloses two separate regions of water being divided by surfactant controlled bilayers and wrapped into a three dimension, periodic, and minimal surface, forming a strongly packed structure [126]. They consist of honeycombed (cavernous) structure and they appear like dots which are slightly spherical in structure. They exhibit size range from 10 to 500 nm in diameter. They have ability to encapsulate hydrophilic, hydrophobic, and amphiphilic substances. Cubosomes have relatively simple preparation methods; they render bioactive agents with controlled and targeted release, possess lipid biodegradability, and have high internal surface area with different drug-loading modalities [127, 128]. Cubosomes are an attractive choice for cosmeceuticals, so for this reason a number of cosmetic giants are investigating cubosomes. Various patents have been filed regarding the cosmetic applications of cubosomes.

## 3. Major Classes in Nanocosmeceuticals

**Figure 12:**
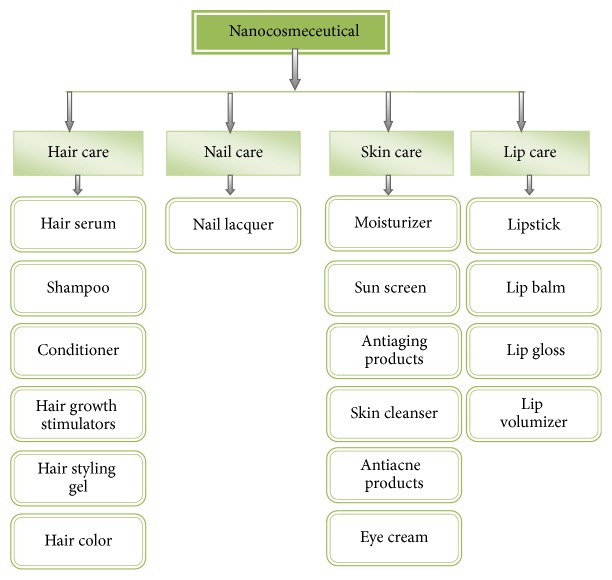
Major classes in nanocosmeceuticals.

Cosmeceuticals are contemplated as the fastest growing segment of personal care industry. A plethora of nanocosmeceuticals are assimilated in nail, hair, lip, and skin care. Major classes in nanocosmeceuticals are depicted in Figure 12 [48].

### 3.1. Skin Care

Cosmeceuticals for skin care products ameliorate the skin texture and functioning by stimulating the growth of collagen by combating harmful effect of free radicals. They make the skin healthier by maintaining the structure of keratin in good condition. In sunscreen products zinc oxide and titanium dioxide nanoparticles are most effective minerals which protect the skin by penetrating into the deep layers of skin and make the product less greasy, less smelly, and transparent [129]. SLNs, nanoemulsions, liposomes, and niosomes are extensively used in moisturizing formulations as they form thin film of humectants and retain moisture for prolonged span. Marketed antiaging nanocosmeceutical products assimilating nanocapsules, liposomes, nanosomes, and nanospheres manifest benefits such as collagen renewal, skin rejuvenation, and firming and lifting the skin [130].

### 3.2. Hair Care

Hair nanocosmeceutical products include shampoos, conditioning agents, hair growth stimulants, coloring, and styling products. Hair follicle, shaft targeting, and increased quantity of active ingredient are achieved by intrinsic properties and unique size of nanoparticles. Nanoparticles subsuming in shampoos seals moisture within the cuticles by optimizing resident contact time with scalp and hair follicles by forming protective film [131]. Conditioning nanocosmeceuticals agents have purposive function of imparting softness, shine, silkiness, and gloss and enhance disentangling of hair. Novel carriers like niosomes, microemulsion, nanoemulsion, nanospheres, and liposomes have major function of repairing damaged cuticles, restoring texture and gloss, and making hair nongreasy, shiny, and less brittle [132].

### 3.3. Lip Care

Lip care products in nanocosmeceuticals comprise lipstick, lip balm, lip gloss, and lip volumizer. Variety of nanoparticles can be coalesced into lip gloss and lipstick to soften the lips by impeding transepidermal water loss [20] and also prevent the pigments to migrate from the lips and maintain color for longer period of time. Lip volumizer containing liposomes increases lip volume, hydrates and outlines the lips, and fills wrinkles in the lip contour [133].

### 3.4. Nail Care

Nanocosmeceuticals based nail care products have greater superiority over the conventional products. The nail paints based on nanotechnology have merits such as improved toughness, fast dryness, durability, chip resistance, and ease of application due to elasticity [134]. New strategies such as amalgamating silver and metal oxide nanoparticles have antifungal properties in nail paints for the treatment of toe nails due to fungal infections [135].

## 4. Toxicity of Nanoparticles Used in Cosmeceuticals

Number of workforce and customers exposed to nanoparticles are escalating because of increasing production and application of the wide diversity of cosmeceuticals products that contain nanomaterials. Despite their huge potential benefit, little is known about the short-term and long-term health effects in the environment and organisms. Due to health hazards, product functionality, and environmental concerns, there may be possible constrains. Concerns have been raised on the possible dangers which may arise on the skin penetration of nanomaterials after their application to the skin [136].

Toxicity of nanoparticles immensely depends on variety of factors like surface properties, coating, structure, size, and ability to aggregate and these factors can be altered and manipulated in the manufacturing process. Nanoparticles having poor solubility have been shown to cause cancer and can exhibit more pronounced toxicity [137]. Health hazard may arise due to the surface area of nanoparticles when compared with the same mass concentration of large particles. Toxicity also depends on the chemical composition of nanoparticles which is absorbed on the skin [138]. There is a relationship between particle size and toxicity: the smaller the size of the nanoparticles, the greater the surface area to volume ratio, due to which there is higher chemical and biological reactivity.

**Figure 13:**
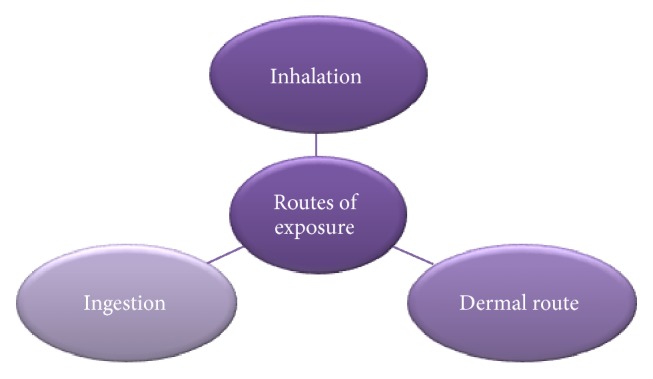
Routes of exposure of nanoparticles.

Health hazard caused by nanoparticles to the humans depends on the degree of exposure and the route through which they access the body. Inhalation, ingestion, and dermal routes are the possible routes by which humans can get exposure to the nanoparticles [139]. Routes of exposure of nanoparticles are given in Figure 13.

### 4.1. Inhalation

According to the National Institute of Occupational Health and Safety, the most common route for exposure of airborne nanoparticles is inhalation. Consumers may inhale the nanoparticles and may get exposure through respiratory route, while consuming the products, such as perfumes, powders, and aerosol and workers can get exposed to the nanoparticles during the production. Evidences from the studies conducted on animals suggest that vast majority of nanoparticles inhaled enter the pulmonary tract and some may travel via nasal nerves to the brain and gain access to other organs via blood [140]. Silicon dioxide inhalation toxicity study suggests that particle size of 1–5 nm produces more toxicological response than 10 nm equivalent dose. Experiments on carbon nanotubes have revealed that on chronic exposure interstitial inflammation and epithelioid granulomatous lesions are caused in lungs. Some carbon based fullerenes might oxidize cells or may be hazardous when inhaled [141]. Results of pulmonary administration of TiO_2_ ultrafine particles than TiO_2_ fine particles show that ultrafine particles resulted in more lung injury. Gold nanoparticles of sizes 2, 40, and 100 nm, when exposed to intratracheal route, were found in the liver and macrophages. It has been demonstrated that the exposure to TiO_2_ of particle size 20 nm even at low doses causes complete destruction of DNA, whereas 500 nm TiO_2_ have small ability for DNA strand breakage [142].

### 4.2. Ingestion

Nanomaterials may be ingested in the body from unintentional to intentional transfer from hand to mouth. Nanoparticles can be ingested from cosmeceuticals that are applied on lips or mouth like lipsticks, lip balms, lip gloss, and so on [143].

According to the studies, after ingestion, nanomaterials rapidly pass out of the body but sometimes some amount may be taken up that can migrate to the organs. Studies conducted on the layers of pig skin show that certain nanomaterials can penetrate in the layers of the skin within 24 hours of exposure [144]. When mice were orally ingested with zinc oxide nanoparticles with 20 nm and 120 nm at different doses, as a result spleen, heart, liver, bones, and pancreas became the target organs. Copper nanoparticles are found in variety of commercially available cosmeceuticals. Mice exhibited toxicological effects and heavy injuries to internal organs when exposed to copper nanoparticles [145]. Silver nanoparticles are widely used in wound dressing and antimicrobial formulations and now are being used in cosmeceuticals like soaps, face creams, and toothpaste. Silver nanoparticles are used for their antimicrobial activity in the cosmeceuticals. The silver concentration that is lethal for bacteria is the same concentration which is lethal for both fibroblasts and keratinocytes [146]. Various studies conducted on rats suggest that silver nanoparticles when exposed to rat neuronal cells led to size decrease and irregularities in shape and also demonstrated that mouse germline stem cell even at low concentrations of silver nanoparticles reduced drastically the function of mitochondria and cell viability. When mice were exposed to gold nanoparticles of 13.5 nm by ingestion, significant decrease in the RBCs, body weight, and spleen index was observed [147].

### 4.3. Dermal Routes

Intracellular, transcellular, and transfollicular are the three pathways by which infiltration across the skin occurs. The dermal exposure of lesser size particles <10 nm can penetrate more easily and are disastrous than greater ones >30 nm. There are possibilities that nanoparticle penetration could be affected by skin barrier alterations such as scrapes, wounds, and dermatitis conditions [148]. Prolonged erythema, eschar formation, and oedema were reported with nanoparticles less than 10 nm. Fullerenes are currently being used in cosmeceuticals like moisturizers and face creams but the toxicity related to them remains poorly understood. Report by Professor Robert F. has identified that face creams which have fullerenes incorporated are found to cause damage in the brain of the fish and have toxic effects in the liver cells in humans [149]. Some studies demonstrated that fullerene-based peptides had the capability of penetrating intact skin and their traversal into dermis could be easy due to mechanical stressor. Quantum dots taken intradermally could penetrate regional lymph nodes and lymphatics. There are proven studies that engineered nanoparticles like single or multiwall carbon nanotubes, quantum dots with surface coating, and nanoscale titania are able to alter gene or protein expression and have lethal effects on epidermal keratinocytes and fibroblast [150]. Currently there are few issues regarding the impact of nanoparticles of titanium dioxide and zinc oxide in sunscreens on health, safety, and environment. Increased production of reactive oxygen species (ROS), including free radicals, is due to greater surface area, greater chemical reactivity, and smaller size. Free radical and reactive oxygen species production is the primary mechanism for toxicity of nanoparticles. Titanium dioxide and zinc oxide generate ROS and free radical when exposed to ultraviolet (UV) radiations, which have potential in inflammation and oxidative stress and can significantly damage membranes, proteins, RNA, DNA, and fats within cells [151]. A research on TiO_2_ nanoparticles toxicity demonstrated that when these nanoparticles subcutaneously given to the pregnant mice, they were transferred to the offspring and there was a reduced sperm production in male offspring and brain damage as well. Nanoparticles of cobalt-chromium have potential that they can cross the skin barrier and damage fibroblast in humans [152].

## 5. Global Scenario of Nanocosmeceuticals

Drugs are subjected to the stringent scrutiny requirements imposed by FDA for their approval but there are no such requirements for cosmetics. Cosmeceuticals are the products which are on the borderline between cosmetics and pharmaceuticals. The Federal Food, Drug and Cosmetics Act and FDA do not recognize the term “cosmeceuticals” and the aesthetic and functional benefits are enjoyed by the products without crossing over into becoming over the counter drugs [153]. Many cosmeceuticals alter the physiological processes in the skin, but manufactures avoid holding clinical trials and making the specific claims to avoid subjecting their products to expensive and lengthy approval process by FDA. New and unfamiliar challenges are being faced by the cosmetic industry [154].

Extra category is created by some jurisdiction to accommodate cosmeceuticals or borderline products.

*Japan*. The products that fall between cosmetics and drugs are called “quasi-drugs.” Ingredients must be preapproved in the market before including them into the quasi-drugs and require preapproval before selling them in the market [155].

*Korea*. Cosmeceuticals are classified as “functional cosmetics” by Korea Food and Drug Administration (KFDA). For improving safety and evaluation of functional cosmetics KFDA is responsible [156].

*Thailand*. According to the ingredients used in cosmeceuticals, they are classified as “controlled cosmetics.” Before being marketed in Thailand, controlled cosmetics require controlled ingredients that require the notification from FDA for the use of products.

*New Zealand*. The category in which cosmeceuticals are accommodated is called “related products.”

*Australia*. In Australia goods can be categorized on the basis of claims about the product and product composition; the borderline products are classified as “therapeutic goods.” Only approved ingredients are used for the manufacturing of these products. Australian Register of Therapeutic Goods registers the therapeutic goods [157].

*Canada.* Cosmeceuticals are termed as “dermo-cosmetics” in Canada. Cosmeceuticals are not recognized as an independent cosmetic category; Canadian health authorities have identified Category V to accommodate products falling in category of both cosmetics and drugs. Less regulatory requirements are required for regulation of these products.

*USA*. There are 3 categories in US: namely, cosmetics, drugs, and OTC drugs, and there is no legal definition of cosmeceuticals according to USFDA. Classification in USFDA depends on the claims of the products [158].

*European Union*. In European Union, cosmetics are regulated under Cosmetic Directive 76/768/EEC. EU does not have category to be called cosmeceuticals, but it has stringent laws in which any claims made by the company are required to be submitted as a proof. According to new regulation by EU, manufacturers have to list the nanoparticles contained in the product which has to be marketed with European Union. Cosmetic regulation states that any product containing nanomaterials as ingredient should be clearly mentioned and has to insert word “nano” in brackets after ingredient listing [159, 160].

*China*. Cosmeceuticals are regarded as “cosmetics for special use.” According to China Food and Drug Administration (CFDA), all foreign cosmetic product manufacturers before selling the product in the Chinese market must complete a safety and health quality test and obtain a hygiene permit. Special use cosmetics have to undergo safety and health quality test such as microbiology, toxicology test, chronic toxicity, carcinogenic test, and conducting safe-for-human-use trials. Imported cosmetics are classified into two categories: ordinary cosmetics and special use cosmetics. Each category of cosmetics requires different type of license from State Food and Drug Administration (SFDA). For the marketing of cosmetics hygiene license or record-keeping certificate from Health Administration Department of the State Council-SFDA must be obtained [161].

If the FDA finds out that there is safety issue regarding use of any cosmetic or the ingredient including nanoparticles, FDA has authority to prohibit the sale and manufacturing of the product or various other options like ban on ingredients, seizing unsafe products, warning letters, and mandatory warning labels and even ban of the product worldwide. New research strategy has been issued by the US Environmental Protection Agency (EPA) so as to proactively examine the impact on environment and human health due to nanoparticles being used in cosmetics, sunscreens, paints, and so on [162]. Under this agency, focus is on the research on seven types of manufactured nanomaterials: titanium dioxide, silver nanoparticles, nanotubes, cerium oxide, fullerenes, and zero-valent [163].

Scientific committee on Consumer Products (SCCP) has raised concern over use of insoluble nanoparticles used in cosmetics that are applied topically because of the toxicity reasons. The royal society world's oldest scientific organization has also raised questions on nanoparticles whether it could enter the bloodstream, taken up by cells and impart its effect [164]. Simultaneously it has also expressed the desire for the conduction of more research in this field to address the chronic effects which may arise as a result of long-term use by people all over the globe [165].

## 6. Conclusion

Nanotechnology is considered to be the most promising and revolutionizing field. Over the last dozen of years, nanotechnology is widely being used and is beneficial in the field of dermatology, cosmetics, and biomedical applications as well. New technologies and novel delivery systems have been invented by scientists, which are currently being used in the manufacture of cosmeceuticals. By the increase in use of cosmeceuticals, the conventional delivery systems are being replaced by the novel delivery systems. Novel nanocarriers which are currently being used are liposomes, niosomes, NLC, SLNs, gold nanoparticles, nanoemulsion, and nanosomes in various cosmeceuticals. These novel delivery systems have remarkable potential in achieving various aspects like controlled and targeted drug delivery, site specificity, better stability, biocompatibility, prolonged action, and higher drug-loading capacity. There is lack of convincing evidences for the claims of effectiveness, so industries are required to provide them. There are huge controversies regarding the toxicity and safety of the nanomaterials; various researches are being carried out to determine the possible health hazard and toxicity. Meticulous studies on the safety profile of the nanomaterials are required. Nanoproducts should be fabricated in such a way that their value and health of the customers are improved. Clinical trials are not required for the approval of cosmeceuticals so the manufacturers enjoy the benefit and avoid holding clinical trials and lengthy procedures. Lastly, stringent laws should be imposed on the regulation and safety of cosmeceuticals and nanoparticles used in them.

## Figures and Tables

**Table 1:** Marketed formulations of liposomes.

Product name	Marketed by	Uses
Capture Totale	Dior	Removes wrinkles and dark spots and has radiance effect with sunscreen
Dermosome	Microfluidics	Moisturizer
Decorte Moisture Liposome Face Cream	Decorte	Moisturizer
Decorte Moisture Liposome Eye Cream	Decorte	Moisturizes, firms, and brightens the delicate skin around the eyes
Natural Progesterone Liposomal Skin Cream	NOW Solutions	Maintenance of healthy feminine balance
C-Vit Liposomal Serum	Sesderma	Hydration, boosts collagen synthesis, enhances skin's elasticity and firmness, and brightens the complexion
Advanced Night Repair Protective Recovery Complex	Estée Lauder	Skin repair
Fillderma Lips Lip Volumizer	Sesderma	Increases volume of lips, fills wrinkles contour, moisturizes the skin, and outlines the lips
Lumessence Eye Cream	Aubrey Organics	Antiwrinkle & firming
Russell Organics Liposome Concentrate	Russell Organics	Hydrating & rejuvenating. Makes skin firmer, softer, and smoother
Clinicians Complex Liposome Face & Neck Lotion	Clinicians Complex	Nourishes skin and prevents photoaging
Kerstin Florian Rehydrating Liposome Day Crème	Kerstin Florian	Moisturizer

**Table 2:** Marketed formulations and uses of niosomes.

Product name	Marketed by	Uses
*Niosome+ Perfected Age Treatment *	Lancome	Removes wrinkles
*Niosome+*	Lancome	Foundation cream, clear and white skin tone
*Mayu Niosome Base Cream*	Laon Cosmetics	Whitening and moisturizing
Anti-Age Response Cream	Simply Man Match	Treatment of wrinkles
*Identik Masque Floral Repair*	Identik	Hair repair masque
*Identik Shampooing Floral Repair*	Identik	Hair repair shampoo
*Eusu Niosome Makam Pom Whitening Facial Cream*	Eusu	Skin whitening

**Table 3:** Various marketed formulations of solid lipid nanoparticles.

Product name	Marketed by	Uses
Allure Body Cream	Chanel	Body moisturizer
Allure Parfum Bottle	Chanel	Perfume
Allure Eau Parfum Spray	Chanel	Perfume
Soosion Facial Lifting Cream SLN Technology	Soosion	Antiwrinkle cream
Phyto NLC Active Cell Repair	Sireh Emas	Skin rejuvenation, overcomes hyperpigmentation, moisturizing & skin firming

**Table 4:** List of marketed products, manufacturers, and uses of NLC.

Product name	Marketed by	Uses
Cutanova-Cream Nanorepair Q10	Dr. Rimpler	Smoothing of fine lines, promotes restructuring of skin & aging
Intensive Serum Nanorepair Q10	Dr. Rimpler	Antiwrinkle serum, fights sign of aging
Cutanova Cream Nanovital Q10	Dr. Rimpler	Antiaging treatment with UV-protection, protective,
Iope Supervital Extra Moist Softner	Amore Pacific	Moisturizes dry and rough skin
Iope Supervital Extra Moist Eye Cream	Amore Pacific	Removes eye wrinkles, dullness, and poor elasticity
Surmer Masque Crème Nano-Hydratant	Isabelle Lancray	Restricting dry and dehydrated skin, reduction of wrinkles and fine lines
Olivenöl Augenpflegebalsam	Dr. Theiss/Medipharma cosmetics	Removes wrinkles, eye rings, and swelling of eyes
Olivenol Anti Falten Pflegekontrat	Dr. Theiss/Medipharma Cosmetics	Antiwrinkle and skin tightening
Regenerations Cream Intesive Ampoules	Scholl	Promotes cell regeneration and smoothes wrinkles
Swiss Cellular White Illuminating Eye Essence	La prairie	Removes under eye darkness and discoloration
Surmer Crème Legère Nano-Protection	Isabelle Lancray	Intensely hydrating

**Table 5:** Various marketed formulations of nanoemulsion.

Product name	Marketed by	Uses
Korres Red Vine Hair Sun Protection	Korres	Prevents hair color from fading away

Nanocream	Sinerga	Wet wipes

Vital Nanoemulsion Α-VC	Marie Louise	Nutrition and miniaturization

Bepanthol-Protect Facial Cream Ultra	Bayer HealthCare	Moisturizing, antiaging, and antipollution

Coco Mademoiselle Fresh Moisture Mist	Chanel	Prolongs fragrance effect

Precision-Solution Déstressante Solution Nano Émulsion Peaux Sensitivity	Chanel	Moisturizer

Coni Hyaluronic Acid & Nanoemulsion Intensive Hydration Toner	Coni Beauty	Skin hydration

Phyto-Endorphin Hand Cream	Rhonda Allison	Softens and smoothes the skin

Nanovital Vitanics Crystal Moiture Cream	Vitacos Cosmetics	Skin moisturizing, elastic, and lightening effects

Vitacos Vita-Herb Nona-Vital Skin Toner	Vitacos Cosmetics	Moisturizer

**Table 6:** List of marketed formulation of gold nanoparticles.

Product name	Marketed by	Uses
Chantecaille Nano Gold Energizing Cream	Chantecaille	Revitalizes, stimulates cell regeneration, collagen production, helps sun damage repair, and makes skin firm

Chantecaille Nano Gold Enerizing Eye Serum	Chantecaille	Prevents aging, promotes collagen, reduces inflammation, and cell growth repair

Ameizii Nano Gold Foil Liquid	Ameizii	Repairs skin damage, moisturizes, and promotes skin whitening

LR Nano Gold Day & Silk Day Cream	LR Zeitgard	Protects skin from UV rays and prevents light-induced premature skin aging

Nuvoderm Nano Gold Anti-Aging Lifting Serum	Nuvoderm	Reduces appearance of signs of aging including fine lines and wrinkles. Promotes collagen & elastin production

Orogold 24K Nano Ultra Silk Serum	Orogold	Restores loss of moisture, improves wrinkles and fine lines, and maintains healthy skin

Tony Moly Nano Gold BB Cream SPF 50 PA+++	Tony Moly	Skin whitening, decreases wrinkles, and blocks UV rays

O3+ 24K Gold Gel Cream	O3+	Makes skin glow and shine

**Table 7:** Marketed formulation of nanospheres [10].

Product name	Marketed by	Uses
Hydralane Ultra Moisturizing Day Cream	Hydralane Paris	Deep moisturizing and hydration

Fresh As A Daisy Body Lotion	Kara Vita	Moisturizing body lotion

Lip Tender	Kara Vita	Lip moisturizer

Nanosphere Plus	Dermaswiss	Antiaging and antiwrinkle

Coryse Salome Competence Hydration Ultra-Moisturizing Cream	Coryse Salome Paris	Moisturizing cream

Eye Tender	Kara Vita	Antiwrinkle

Nano Saltmmoisture Key	Salvona	Moisturizer

Clear It! Complex Mist	Kara Vita	Antiacne

Cell Act DNA Filler Intense Cream	CellAct Switzerland	Reduces wrinkles and firms skin
